# An Automatic Aggregator of Power Flexibility in Smart Buildings Using Software Based Orchestration

**DOI:** 10.3390/s21030867

**Published:** 2021-01-28

**Authors:** Dharmendra Sharma, Jari Rehu, Klaus Känsälä, Heikki Ailisto

**Affiliations:** VTT Technical Research Centre of Finland, Kaitoväylä 1, 90570 Oulu, Finland; jari.rehu@vtt.fi (J.R.); klaus.kansala@vtt.fi (K.K.); heikki.ailisto@vtt.fi (H.A.)

**Keywords:** smart building automation, energy optimization, energy flexibility, aggregator, networked energy resources, building energy management system, IoT, cyber physical systems

## Abstract

This paper presents a software-based modular and hierarchical building energy management system (BEMS) to control the power consumption in sensor-equipped buildings. In addition, the need of this type of solution is also highlighted by presenting the worldwide trends of thermal energy end use in buildings and peak power problems. Buildings are critical component of smart grid environments and bottom-up BEMS solutions are need of the hour to optimize the consumption and to provide consumption side flexibility. This system is able to aggregate the controls of the all-controllable resources in building to realize its flexible power capacity. This system provides a solution for consumer to aggregate the controls of ‘behind-the-meter’ small loads in short response and provide ‘deep’ demand-side flexibility. This system is capable of discovery, status check, control and management of networked loads. The main novelty of this solution is that it can handle the heterogeneity of the installed hardware system along with time bound changes in the load device network and its scalability; resulting in low maintenance requirements after deployment. The control execution latency (including data logging) of this BEMS system for an external control signal is less than one second per connected load. In addition, the system is capable of overriding the external control signal in order to maintain consumer coziness within the comfort temperature thresholds. This system provides a way forward in future for the estimation of the energy stored in the buildings in the form of heat/temperature and use buildings as temporary batteries when electricity supply is constrained or abundant.

## 1. Introduction

Electricity consumption patterns are on rise worldwide and the share of energy consumption in residential buildings is following the same trend. In 2018, the household buildings’ alone represented 26% of final energy consumption in European Union (EU) wherein renewables accounting for only 20% of final energy consumption in household [1] and overall buildings (including service sector) were responsible for 40% of energy consumption in EU while contributing to 36% of CO_2_ emissions ([2], p. 6). Globally, buildings were responsible for 28% of energy-related CO_2_ emissions (considering indirect emissions from upstream power generation) reaching to all time high of 10 GtCO_2_ in 2019 [3]. Energy use for thermal comfort (space cooling and space heating) has majority share in buildings’ energy use worldwide and it will be responsible for almost half of the new global electricity demand growth in buildings’ energy use by 2050 with share of 37% and 12.4% respectively [4]. To minimize of CO_2_ emissions from buildings, there is an urgent need to promote the local generation and intelligent consumption of more and more renewable energy sources in buildings along with promoting use of renewable load technologies especially for space cooling and space heating.

Moreover, a clean, cheap and continuous electricity supply is also becoming a major challenge for grid operators and other energy stakeholders due to growing energy demand in peak hours and congestion problems due to increasing integration of distributed generations in distribution networks [5]. Therefore, consumption management of thermal comfort technologies at local level is need of hour to enable the controllable, optimized, balanced and shared use of energy. 

Over the years, there are several efforts made to make demand response in residential buildings a viable solution [6]. A BEMS can help to achieve control over building’s loads to optimize the consumption based on indoor environmental parameters [7]. Recently, the demand of BEMS systems is increasing however the scope of BEMS implementation can vary from case to case [8]. A BEMS system can have modularity [9], hierarchical design [10], comfort prioritized building automation [11], smart grid integration possibilities [9], agent-based control [12]. These cited work are good from their own application goal point of view however in order to make high performance buildings the BEMS systems must be robust, modular, and hierarchical along with features of automatic load aggregation, aggregator control access to outside, automatic comfort control, resident’s data protection mechanisms, routines for information exchange, smart bot/smart grid integration possibilities etc. We present such a solution in this paper. The maintenance and operational expenses of BEMS solutions can require several work hours for both hardware and related software work. Our aim is to make a system with low maintenance and customizable features that can be used to manage several building energy resources in flexible or optimal manner along with prioritizing the resident needs. 

Proper understanding of loads and their management is alone very important and therefore, in this work, we will detail and demonstrate the control of different types of loads; leaving the local production, local battery storage and electric vehicle out of scope of this paper. Moreover, this BEMS solution can operate in both hot and cold climates therefore, we justify the need of this solution in both type of climates in Section 1.1 and Section 1.2. Continuing further in this introduction section, we investigate the final use of electricity for thermal comfort by presenting facts and future trends from different geographies and countries. The major challenges are divided in separated sections for space heating (Section 1.1) and space cooling (Section 1.2) to reduce complexities of information presented. However, if reader is familiar with challenges posed by space heating and space cooling sector then reader may jump to Section 1.3 that describes the details of this BEMS system along with its technological design challenges and their solution. 

### 1.1. Space Heating Sector; Overview, Peak Power Challenges and Opportunities in Future

The space-heating sector is slowly transitioning from fossil to renewable energy use and this transition is a must to order minimize CO_2_ emissions in the future. Space heating accounts for a majority share (64%) of final energy use in households in the EU, with renewable energy accounting for only 27% of EU households’ space heating consumption in 2019 [1]. In buildings, heat pumps are a popular substitute of fossil fuel-based heating systems. In fact, a report [13] concludes that in selected EU-21 countries the number of heat pumps reached 11.8 million covering almost 10% of buildings. Furthermore, countries with extreme cold climate conditions are adopting heat pumps at a much faster rate. Finland is a good example of an emerging leader in the adoption of heat pump technology and according to the Finnish Heat Pump Association (SULPU), 98,000 heat pumps were sold in 2019, representing a 30% increase in a year. The total number of heat pump installations have surpassed 1 million in Finland [14]. Heat pumps are an important technology for the transformation of space heating sector because first, heat pumps are energy efficient; on average, space heating using heat pumps is 300% efficient, meaning that, 3 kW of thermal energy is generated by heat pump for each kW for electricity consumed ([2], p. 16, [9,10]). Second, heat pumps are sustainable; energy consumption from electricity mix by replacing fossil fuel-based district heating can help to reduce CO_2_ emissions reduction in different scenarios [2]. 

On the other hand, this increasing installation of heat pumps is responsible for increasingly high peaks in power demand, especially in winter when temperatures are extremely low [15]. The reason for this peak demand is first, they use an electricity mix as energy source and second, the efficiency and capacity of the heat pump decreases along with the decrease in temperature ([2], p. 16). An example of very high peak power demand due to household energy use in Finland, is 7 January 2016 [16] when consumption-weighted outside temperature was −25 °C. According to an estimate [17], this increased household demand was caused by the excessive heating needs along with decreased heat pump efficiency at lower temperature and quantitatively speaking, an additional 100–200 MW of electricity are required in Finland to counter the increased demand due to each 1 °C of outdoor temperature drop. Fischer et al. [18] concludes that a 64% penetration of heat pumps in building stock increases the annual electric demand of the individual household by a factor of 2.8 and the average daily electric demand during the coldest weeks of the year by more than a factor of four. In addition, heat pumps mainly cause the highest peaks in the coldest weeks and at a household level, load peaks are increased up to seven times, due to the use of electric back-up heaters during the coldest days of the year.

On the contrary, if implemented well then heat pump systems can be an opportunity for imminent renewable energy transition along with managing peak power demands. Nowadays, it is widely being recognized that heat pump driven space heating can be the enabler of demand-response in buildings in smart grid friendly way [19,20]. It is also proven that controllable heat pump pool can be a great enabler for intelligent consumption (“part-time for part-space”) of electricity to thermal energy resulting in peak power reduction and shifting in order to make grids truly smart. According to results (of simulated pool of heat pumps) presented by Fischer et al. [21], the pool can provide high shift-able energy and high load shifting efficiency. In case of air source heat pumps, short term flexibility (up to couple of hours) can be realized by means of stored energy in thermal mass [22,23,24] of the well-insulated building (energy released in a non-controllable way). 

### 1.2. Space Cooling Sector; Overview, Peak Power Challenges and Opportunities in Future

Energy requirements for space cooling are also increasing at an alarming rate worldwide. In absolute terms, space cooling was responsible for 8.5% of total global electricity consumption and emissions of about 1 GtCO_2_ in 2019, almost tripling since 1990 [25]. It is also the fastest growing end use of electricity in buildings [25] and the use of air conditioners (ACs) and cooling fans accounts for 20% use of energy in buildings [4]. Emerging market countries are now leading this space cooling technology use race.

The unplanned installation and use of ACs has similar repercussions on power systems, as heat pumps. According to a worldwide estimation by the International Energy Agency (IEA) the AC global stock in buildings will grow to 5.6 billion by 2050 covering 2/3 of world’s households, up from 1.6 billion in 2016 since most households in tropical countries are yet to install their ACs [4]. For example, in 2018 household ownership of ACs in the USA and Japan is above 90%, whereas in India, it is only 5% and growing with 15% each year. It there are no improvements in the energy efficiency of ACs despite the global efforts to promote efficient cooling [26] then the global space cooling energy demand will grow due to the new AC installations in the future, more than tripling by 2050 [4]. Moreover, consumers also tend to buy cheaper, less efficient AC equipment due to the higher up front cost as per reports on Indian [27] and Chinese [28] AC consumer buyer behavior. This soaring worldwide demand for inefficient ACs will complicate the decarbonization of grids. This issue of space cooling is more sensitive in fast growing countries; in China space cooling accounted for 16% of the peak electricity load in 2017 and it can reach up to 50% of peak electricity demand on extremely hot summer days, as observed in recent years [29]. Although the share of ACs in the peak power demand is lower at 10% in India due to the low AC penetration, the biggest increase in power peaks is also happening in Indian cities. In Delhi, space cooling consumes more than 50% of the total annual electricity [29] and a temperature increase of 1 °C increases the demand in Delhi by approximately 120 MW [30]. Without any actions, the share of ACs in peak electricity demand could reach up to 45% in 2050 [31].

On the contrary, if implemented well then air-conditioners (ACs) can be an opportunity to maximize the consumption of locally generated renewable energy along with managing power peaks and maximizing the comfort of residents in hot climates. The smart grid-assisted control and management of large pools of ACs could help the future electricity systems handle the peak power problems. A buildings with well-maintained ventilation system (preferably with heat exchangers) and “part time” for “part space” space cooling control mode is beneficial solution at so many levels through controllable use of energy. The flexibility potential of AC systems in residential buildings is also significant in buildings [32] yet in order to properly utilize it in profitable way certain aspects such as number of AC units in pool, outdoor weather conditions, required comfort level, building size, building structure and orientation with sunlight need to be considered.

### 1.3. Solution for Increasing Thermal Demand Peaks Due to Buildings

The peak demand can be very expensive to manage for energy suppliers, due to imbalanced costs, if their day ahead predictions are not accurate. This issue can be managed efficiently if there are control tools available to reduce/shift the peak demand. In order to maintain the power system balance, the grid sourced energy consumption needs to be changed at consumer sites in real time following the abrupt changes in the energy production and residential buildings can help to do that up to some level. This digitization of power systems’ is happening and it is enabling the energy consumers to participate in local energy markets through active load regulation, use of behind-the-meter generations and battery storage. Presently only 2% of global potential for demand-side flexibility is being utilized [33]; presently it is limited to big flexibility sources but new automation tools to aggregate the flexibility of several small scale loads can result in better control and use of local energy resources while increasing the overall systems’ flexibility. In addition, digitalization and smart demand-side management can reduce overall energy use in buildings by as much as 10% [34]. 

Although an individual household is a small electricity consumer but in order to make ‘future-proof’ energy grids, some of these small electricity consumers will have to play a critical role in future to make grids ‘truly smart’ by first, becoming prosumers by installing local production for partial self-consumption. Second, sharing the energy and energy flexibility locally with other consumers through fair market mechanisms. This shared use of energy is possible by providing the necessary energy flexibility information to all energy stakeholders (including other consumers), and acting in real time to reduce or increase the energy use to help in balancing the grids. Current information and communication technologies (ICT) hardware and flexibility market structures do not enable large-scale utilization of small resources in smart households, and no applicable (generally accepted) standards exist [35,36]. So far, buildings (both residential and service sector) are considered a complicated component of smart-grid environment due to the following facts:

- Unavailability of control access to ‘behind-the-meter’ loads,
- Heterogeneity of energy consumption loads,
- Unforeseen behavior of inhabitants,
- Safety concerns of residents,
- Privacy concerns of residents and
- Unwillingness of residents to install BEMS solutions due to low return on investment.

All above factors are the reason that buildings are considered blind-spots of smart grid environment and despite the existence of a large smart meter infrastructure in some countries (for example Finland), real time demand response at scale is not a reality yet. In addition, the impact of flexibility of a single device (heat pump or AC or other household appliance) on the overall power systems’ flexibility is very small and therefore barely utilized in practice. Moreover, minimum flexibility requirements for market participation, flexibility variations throughout the year, reliability and complexity of flexibility solutions are other known factors to impede the use of demand side flexibility. For these reasons, aggregation of flexibility at the building level is necessary in order to make the small-scale flexibility trading economical. Meaning that, there is a need scalable and flexible control tools to control the electricity consumption loads in buildings in short response time and a possibility to offer this flexibility to marketplace at very low cost.

The previously stated concerns about buildings are also the reason why a majority of third party flexibility aggregators are not very keen on utilizing the flexibility available from residential consumers; these consumers hold the key to large potential of residential thermal flexibility but they lack the skills and tools to control, manage and trade the energy flexibility as a commodity. Among all energy stakeholders, it is technology developer’s and energy distributor’s major interest to enable the market participation of the small scale electricity consumers by providing matured hardware technologies for aggregated control of their loads and software tools to actuate the controls in short response time by enabling the communication over internet.

Looking at the big picture, there is no technological lack of either the suitable hardware to make wireless controls of consumer loads or the software to manage them. In addition, market of flexibility is growing, the impact of the flexibility of aggregated load resources on power system is already proven [18,21] and new mechanisms to trade the flexibility locally are being developed for example FLEXIMAR marketplace [37]. Therefore, the question arises; what is stopping us to develop tools to utilize the residential flexibility at large scale? Nothing in general, but such automated and scalable tools need great curation to put different software and hardware components together and built over them. In addition, the interest of energy stakeholders and consumers, to promote such technologies, is varying case by case. Besides, it is apparent that there are existing tools and technologies that can be combined with new methodologies to solve the flexibility related challenges associated with buildings as explained below one by one:

- Several loads can be controlled and measured wirelessly though controllable wall plugs [38] (z-wave based hardware in our case, a widely accepted home automation technology),
- Software based tags can be used to describe the type and nature of loads (i.e., description of heterogeneity),
- Energy consumption behavior of inhabitants can be understood with time,
- Software based safety measures can be taken to avoid control of devices beyond user comfort temperature limits,
- Data can be collected, processed, analyzed (later can be destroyed) on premise gateway without sharing the much details with outside world,
- Selling the energy flexibility can result in some extra money for sellers, which is paid by buyer stakeholders in exchange of flexibility.

Finally, in forthcoming sections of this paper, we will discuss methodology in Section 2. Further, hardware and software description are detailed in Section 3 and Section 4, respectively. In the end, we will conclude the work by presenting the results, demo outputs and future work possibilities.

## 2. Methodology

**Figure 1:**
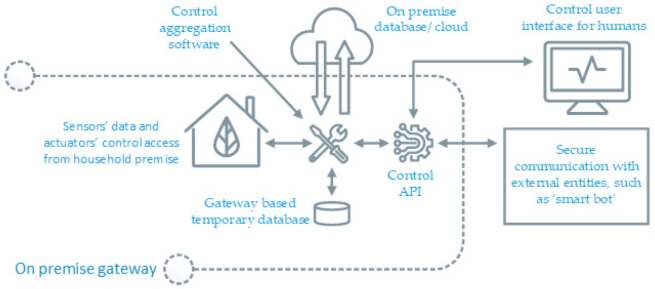
A concept block diagram for aggregation of all actuator hardware controls on premise-based internet enabled gateway computer.

From a technology point of view, the flexibility from residential consumers is a viable option only if the consumption control tools and services are cost effective as well as outstandingly automated for all willing participants. In addition, the demand of the energy flexibility will be more if it is available in sizable chunks in order to be easily tradable. The flexibility offerings of complete household building at once is a better option instead of individual device’s flexibility. Our solution can make a household seen as single aggregated load with queriable up-down flexible power limits. It is implemented by creating local controls of all actuator devices and then making a medium for two-way M2M control communication with external world as depicted in Figure 1. 

It can operate heating/cooling systems based on calculation (of comfort and profitability) to provide flexibility within safety limits instead of force control. As per our setup, every actuator should have one paired sensor in same room to monitor the impact of actuator’s energy use (a temperature sensor for heating or cooling devices, a humidity sensor for humidifiers, a motion sensor for lights and two sensors i.e., CO_2_ and air pressure difference for ventilation equipment monitoring). This system can maintain the temperature, humidity, CO_2_ and air-pressure difference levels in human comfort zone by turning suitable devices on and off. Nevertheless, we only use heating and cooling devices for thermal flexibility since power flexibility from humidifiers, lights and ventilation are not significant in the first place and second, these devices are very critical in their use applications. Therefore, we stick to use of only thermal flexibility in households and we consider other devices as critical, leisure use but our system can operate them in optimal or scheduled manner instead of flexible.

**Figure 2:**
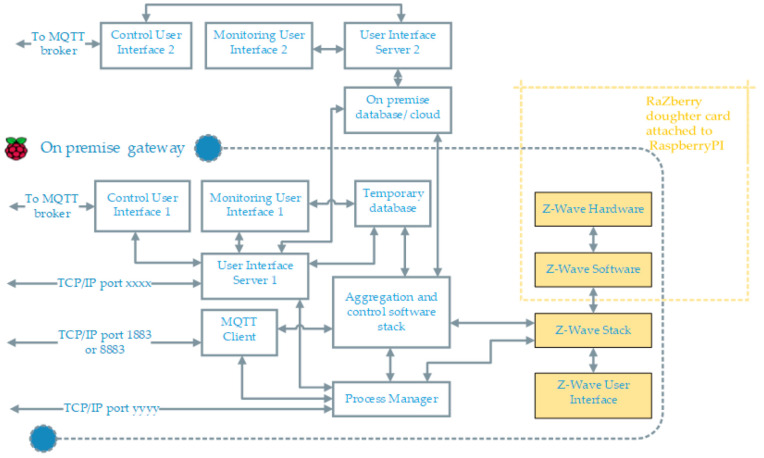
The overall setup and interconnections of hardware and software components (this gateway is GW3 in Figure 3).

In order to have any type of automation of distributed hardware elements at scale, a networked hardware environment is a preliminary requirement to assign unique identities to the elements in that network. We use a z-way RaZberry shield [39] on the top of Rpi gateway. This z-wave transreceiver module along with z-wave software stack maintains the network topology for the added elements in the hardware network. The block diagram of overall system is demonstrated in Figure 2. The ‘aggregation and control software stack’ uses z-wave stack to discover installed actuators and sensors, generate control instances with API control routines enabled; more details are listed in Software Description section. It use MQTT client to receive external control command. The control signal over MQTT is sent either using smart bot or manually using a control user interface. Depending on the use case, we can choose to host the control and monitoring user interface server either on gateway itself or on a different machine. There is also an on-board process manger to monitor, start, stop and diagnose the essential processes remotely. 

Overall, the novelty of this solution is that it is a bottom to top solution with evolutionary nature allowing run time changes in hardware and software system components and requires minimum level of human intervention to further adept the changes in overall setup. This solution alters the electricity consumption to maintain the thermal comfort in residential buildings and it can work in both cold and hot climate conditions controlling numerous type of household devices with the help of suitable hardware add-ons. It also allows us to remote monitoring and control along with aggregation or segregation of controls based on the device type and location within the households.

## 3. Experimental Setup

For flexible control, we are using z-wave mesh network controller and z-way actuator devices, which are good enough for smart control of devices in short range communication within our smaller 50 m^2^ household apartment area as mapped in Figure 4. Z-wave controllers and devices can wirelessly communicate with each other over distances up to 50–100 ft and with a maximum four hops their extended range is approximately 300 ft. On the downside, z-wave devices can be battery intensive in comparison to other wireless technologies but except for thermostats we are not using any battery operated z-wave sensors; we are using in-house developed VTT’s sensor nodes ([40], p. 245). However, these constraints on z-wave network does not make z-wave ideal to be used in very big buildings and other wireless networked technologies may be a better choice for long-range communication. 

**Figure 3:**
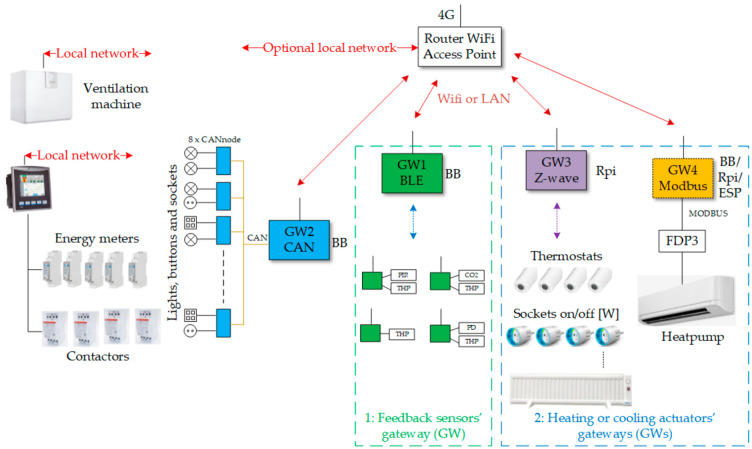
Sensing, metering, computing and communication hardware in residential load site of smart grid laboratory in Oulu.

**Figure 4:**
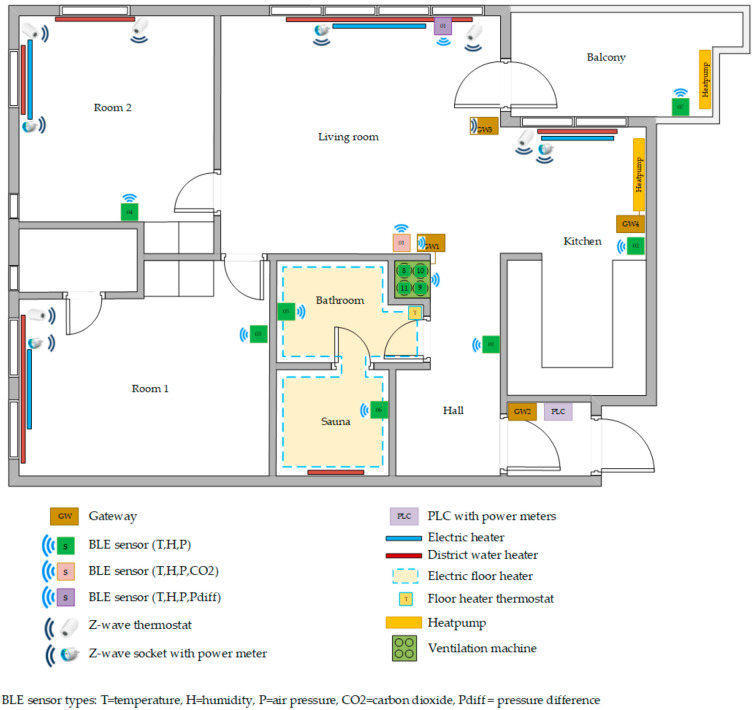
Floor map of residential load site with location of installed devices and ICT component.

The hardware of the test apartment building has been built incrementally in different phases over several years and this residential site is part of smart grid laboratory in Oulu [41]. The apartment load in the smart grid laboratory with every installed hardware is visualized in Figure 3 and the floor map of the site as presented in Figure 4. In the following description section first, we will describe the details of all the hardware devices installed in the apartment and then, we will highlight the hardware that are important for flexible energy use. Currently, the apartment has four gateway (GW); each has its own functionality:

(1) GW1: Read status of indoor condition through wireless sensors and ventilation control,
(2) GW2: Lighting Control, includes a Controller Area Network (CAN) bus interface,
(3) GW3: Flexible heating and cooling control, includes z-wave interface and,
(4) GW4: Heat pump control, includes Modbus interface.

It is important to note that the location of heating equipment, as in Figure 4, should be near to the room windows and the sensor should be far away from windows since in cold countries the area near window is more prone to the effects of outside cold weather. In addition, same logic can used for cooling device installation in hot countries. All collected sensor data from wireless sensors in apartment is written to on premise database. Saved data can be utilized for various visualizations, predictions and for device control in real time. The details of sensing, metering and control hardware are following:

### 3.1. Wireless Sensor Network 

The apartment has 13 indoor sensors. Each room has a basic sensor that measures temperature, humidity and air pressure (THP). In addition, the living room has a CO_2_ sensor, a differential pressure sensor and a motion sensor. The basic THP sensor is also installed in the ventilation tubes. All these sensors work wirelessly and use Bluetooth low energy (BLE) communication to GW1.

### 3.2. Energy Metering (Wired and Wireless)

The power consumption of almost every piece of electrical equipment is measured both separately at an individual level and conjointly at the whole apartment level. The largest loads are measured with meters installed in the electric centre, which are read by a programmable logic controller (PLC) device. The metering measurements of every three-phase and single-phase energy meter are logged to a database through a wired local network. Three phase meters measure main supply, oven and sauna. One phase meters measure heat pump, floor heater, washing machine and some fixed sockets. Smaller loads connected to sockets are also exclusively measured wirelessly with a wall plug meters (Fibaro z-wave, [38]). Therefore, it is possible to determine of energy consumption of different categories and combination of devices easily by having individual and conjoint energy measurements through energy meters at different levels in apartment.

### 3.3. Controllable Devices and Connected Loads

The devices from Figure 3 can be divided into two major categories.

#### 3.3.1. Critical Load Devices

In our setup, lighting and ventilation are considered as critical loads meaning that lighting and ventilation loads are not used to offer any power flexibility; we describe these loads only in brief here. The lighting in the apartment is implemented in 24 V DC. For lighting in the apartment, a CANbus controller is a controllable device and lighting is a load here. In addition, lighting can be a non-critical load in service sector buildings where during times of low occupancy it can be used as a flexible load. The ventilation machinery’s maximum capacity is 75 L/s. This is enough for a maximum of nine persons and normally 5–6 persons on average in the house. 

#### 3.3.2. Non-Critical Load Devices

In our setup, non-critical devices can provide flexibility. The apartment has a central water-heating radiator and an electric heater in each room. In addition, the living room has a heat pump (for both heating and cooling purposes) and the bathroom is equipped with floor heating. All central heating radiators include a (z-wave) thermostat and the set point can be changed wirelessly by control software. All electric heaters are connected via wall plugs through a wireless (z-wave) socket that can be controlled (on/off) and it also measures power consumption of connected load. All functions of the air source heat pump can be controlled by software (on/off, heating, cooling, set point etc.). There is a separate gateway (GW4) for heat pump interface since z-wave shield occupies essential GPIOs of Rpi gateway (GW3). Another reason is that there should be a gateway near to the heat pump to avoid the long Modbus cable. Besides, if we use ESP controller edge with a Modbus interface (for ex. ESP32, [42]) then there is no need of an extra gateway GW4 and the heat pump control also becomes wireless. 

The details of the RaZberry z-way controller and its usages are detailed in its user manual [43]. The description of z-way software stack and its communication with z-way user interface is described in its API documentation [44]. The JavaScript automation engine of z-way controller automatically implement a broad variety of applications using the underlying z-wave devices. As described in Figure 3, in our setup majority of the flexible loads, except heat pump, are controlled by using z-wave devices (i.e., z-wave sockets and thermostats). In practice, apart from managing the z-wave device network topology, the automation engine of z-way controller can also create virtual modules to receive and send data from/to internet-based services or to use other third party technology based on HTTP. As described in Section 2, it is very important for the experimental setup to provide automatic access to all flexible actuators and their feedback sensors though the z-wave software stack. The heat pump and all wireless sensor network devices are based on non-z-wave technology hardware but we have created their proxy virtual ‘functional’ z-way-like devices, in the z-way stack, with unique identities as described in z-way manual ([43], pp. 69–70); making it possible to access all relevant actuators and their feedback sensors by searching through the z-way stack. 

## 4. Software Description

The critical loads should be operated in a way that maximizes residents’ living safety and coziness in households whereas non-critical loads can be operated in two ways: first, in an optimal manner to minimize the electricity use (optimal control) and second, in a flexible manner to provide flexibility services according to the flexible capacity (intelligent flexible control). Our focus in this section is to demonstrate the scalable software architecture of ‘aggregation and control software stack’ for non-critical loads from Figure 3 and emphasize its modular and hierarchical nature. 

### 4.1. Control Software for Critical Loads

As the name implies these devices are important for residents’ health and wellbeing; therefore the controls of critical loads need to be standalone and to be curated with specification of the control device and indoor environment conditions.

The ventilation unit is controlled in a continuously adjustable way (15–100%) based on CO_2_ sensor data. Ventilation keeps the CO_2_ value within the recommended limits. Ventilation also performs an automatic boost if moisture is detected in the bathroom. Ventilation is kept to a minimum level when there is no one in the apartment (away-mode). Based on the motion sensor, the normal mode (home-mode) is set. Both fans of the ventilation machine can be controlled separately. It means that control can compensate for example vacuum caused by cooker hood. Further details of ventilation control can be found in earlier publication ([40], p. 247) related to indoor comfort control of the same apartment building.

The lighting in the apartment is implemented in 24 V DC. All luminaires can be controlled in a continuously adjustable way from the control program. The lighting can also be controlled from standard switches.

### 4.2. Control Software for Non-Critical Loads

As explained in the experimental setup section, it is possible to add all relevant sensors and non-critical loads in z-way software stack in spite of their non z-wave hardware and communication interface. We took advantage of the z-wave network architecture and its software stack to build the control aggregation software. All devices can be assembled under the z-wave stack; devices with a z-wave interface are connected directly via the wireless (z-wave) interface and z-wave non-compatible devices can be connected to the system via the http-rest interface. The z-wave API [45] makes it easy for our aggregation software stack to orchestrate and discover through all networked devices. 

**Figure 5:**
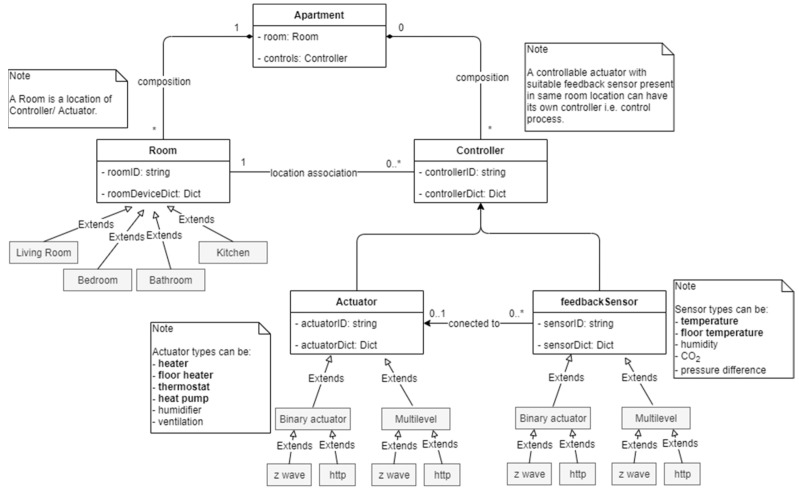
This is a class diagram of automatic aggregation software.

The class diagram of aggregation software (‘aggregation and control software stack’ as in Figure 2) is presented in Figure 5. Here, the question is that why do we need this type of software in the first place? The main motivation for this is to achieve maximum degree of automation in BEMS installation, upgradation or hardware changes. The following goals, achieved by this aggregation software, can further underscore the inspiration behind its development:

- Automatic device discovery (of all sensors, actuator and meters) with help z-way API,
- Automatic instantiation of control process instances for newly added device pairs,
- Every actuator control process is assigned its user defined higher and lower safety threshold limits of indoor conditions,
- Built-in routines to check the trustworthiness (up to some level) of the feedback sensor data (indoor conditions) before acting on external control instruction,
- User defined tag-based prioritization of non-critical loads is also possible and these priority devices are not to be used to serve flexibility,
- Automatic calculation of higher and lower power limits of each actuator and the record of their current power consumption levels,
- Automatic update of aggregated flexible power capacity (both up and down) from all the installed actuators in the apartment building,
- Calculation of total flexible power capacity (both up and down) based on end use (cooling and heating),
- No need of any software configuration in the developed system when a device is added or removed from the z-way network stack,
- Every controller (i.e., control process) is associated with its location in the apartment and the association can be changed if devices are being moved to another location,
- Automatic disabling (later deletion) of control instances if a device is removed.

The essential component of our approach is to integrate context to each element to have their properties, behaviors and location-awareness associated with each device. This can be done by following rules in designing the aggregation software stack:

- Every room with installed heating/cooling equipment, in itself is a closed loop temperature control process with air-temperature set point within the residents’ comfort limits.
- Every actuator should have an associated feedback sensor to monitor the impact of its operation over the time.
- For non z-wave devices, the hardware categories should be specified by using software tags though z-way user interface whereas the z-wave devices already have this feature inbuilt make them identifiable.
- Every actuator and sensor association must have a defined room location.
- The system should shield the user information (i.e., occupant numbers, occupant activity, occupant location, device types, details of power consumption behaviours etc.) from outside world and only share the available aggregated power flexibility if queried.

In order the understand the functionality of the aggregation software in Figure 5, we have to see from practical point of view that there is an apartment building with: (1) many rooms and (2) several pair of actuators and sensors installed within each of those room locations. The ‘Apartment’ class have methods to search through the z-way API and to instantiate all ‘Room’ instances and to find all resources (sensors and actuators) present in every room. This information of resources in the room is kept in form of dictionaries in run time environment and also saved locally in temporary database onboard. After that, the ‘Apartment’ class also instantiates the ‘Controller’ instances by using the resource information from room dictionaries. The information of controller process is kept in run time and also saved locally in temporary database onboard. The list of all controller instances can be segregated by means of priority or flexible defined by user. The external control instruction received by MQTT client is passed to (only after calculation of comfort situation) aggregated flexible control instances. This software is ‘future proof’ to changes made user by in hardware and in device use preferences.

In addition, it allows to receive a M2M control command based on end use of energy (heating and cooling), switch to extreme power levels (minimum or maximum possible power consumption levels), comfort, device category specific control (heater, heat pump, floor heater, thermostat etc.) and room location specific control. In absence of external control signal, the system is fully capable of operating by trying to minimizing the use of electricity and keeping the adequate levels of comfort. It is done by turning on and off loads whenever needed. The system can provide its flexible capacity information to external sources and then, if needed, the flexible control command can be sent by external controlling entity to alter the aggregated loads. The allowed control modes in apartment building are minimum power, maximum power, cooling and heating as detailed in Table 1.

## 5. Results and Conclusions

**Figure 6:**
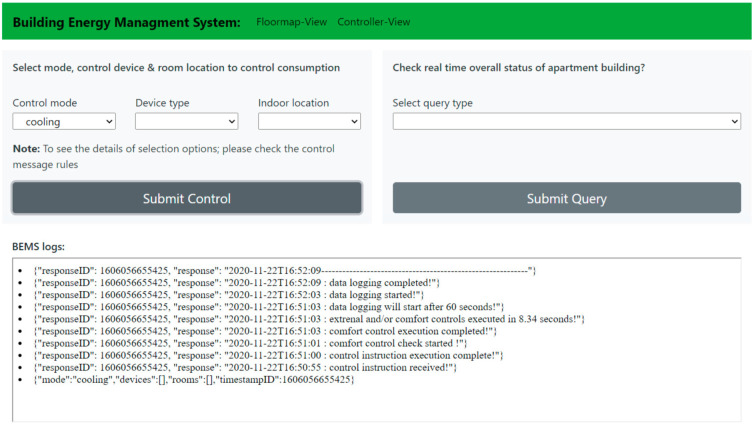
MQTT API-based control user interface for humans.

**Figure 7:**
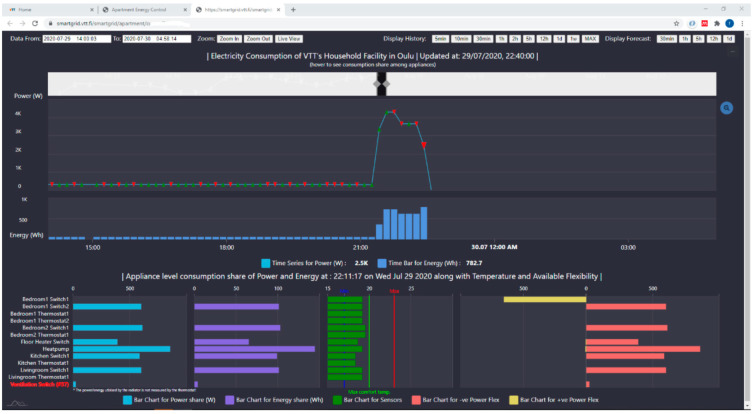
Monitoring user interface.

The paper first shows the overview of increasing thermal demand worldwide and then presents a BEMS solution to tackle challenges of future smart grids with the help of smart buildings. The analysis in Section 1.1 and Section 1.2 clearly indicates that the high performance buildings (in terms of energy consumption and energy flexibility) are a necessity to achieve sustainability goals in built environment. A simple control user interface, of BEMS solution, based on MQTT control API of aggregating software has been shown in Figure 6 and a monitoring user interface, to see the effect of applied control, is displayed in Figure 7. 

The primary result of this BEMS solution is that this concept of modular aggregated control is also scalable; it can understand the different modes and operating power levels of each actuator device and can also alter the mode of operation if appropriate control command is given. Individual control safety loops for each control process are checking comfort level at an interval of 10 min and they will override the external control execution in case the user comfort seems to be compromised. This tool allows the aggregating control along as well as the customized control of actuators based on selected room locations. Moreover, along with on-demand control it can operate in optimized mode to minimize energy consumption or maximize user comfort. The data logging formats for different loads is kept similar irrespective of the type of load or type of energy hardware network. The system can respond to the smart bots querying for up/down power flexibility levels in apartment and comfort status in different room locations and can also give transformer level location information for local energy/energy flexibility trading possibilities.

In addition, the quality of flexible energy service depends on the delivery of constant power level change for promised time duration and it is very important to be to be initiated at promised time instant. The timing of control execution is a variable in our case (therefore important to know) since our setup controls several small loads and when control command is received, they are actuated one by one. The overall actuation time depends on the number of devices in the network and approximately, it is one second for each device. This actuation time (control latency) is time taken in control process status check, control execution, control process status check again and data logging. This control latency is measured on gateway in real time and sent back with control response message as shown in Figure 6. Moreover, for message identification purpose, timestamp based identity is attached to every control or query message sent (a control message identity is necessary for each message and it will be sent back with response). Overall, our system enables top-down authority in control and management of energy resources which is possible by:

(1) scalable and replicable device hardware network,
(2) ‘bottom-top’ hierarchical information network (apartment → room → devices) and methods to support aggregation and/or segregation of information based on different criteria’s (as described in software description),
(3) modular controls and user interfaces built upon the modular information layer.

The developed monitoring interface have historical data visualization with live updates. It enables us to see both big and small details, i.e., energy and power levels at apartment levels and their distribution among individual device levels. Feedback sensor status and up/down flexibility power levels of each device are also displayed. Furthermore, having used the maintainable networked z-wave ICT system enabled the modularity in information, control and visualization layers of the system therefore as a result any changes at device network level are propagating through the systems’ software stack automatically and adopted changes can be seen in monitoring user interface as well. Meaning that, new added or removed devices from physical network are automatically updated in visualization and once deployed no changes in software are needed. 

Conclusively, since the control execution time with data logging takes few seconds therefore in real world applications this BEMS system is suitable where data are updated on at least a minute basis. Moreover, the system’s auto-reconfiguration (re-orchestration of whole device network and update of all control process) time is 30 min therefore if any device is disconnected from network then its control process will be automatically disables after the next auto-reconfiguration and it will be removed from the group of the aggregated flexible loads. In addition, users can define priority software tags to exclude any device (of importance) from the group of the aggregated flexible loads. 

## 6. Discussion and Future Work

Real time measurement and control of power peaks is a necessity for future electricity grids. The fast energy information exchange and control services can help to serve more consumers with existing grid resources by shifting and reducing the power peaks. Secondary ‘behind-the-meter’ wireless energy measurement meters (such as wall socket meter) can minimize the electricity measurement period that can enable the participation of small loads in very short response flexibility marketplaces. 

Here at VTT, we have developed the some pieces of this puzzle i.e.,:

(1) one-tier marketplace for energy flexibility trading, and
(2) automatic aggregator of power flexibility for small loads (as presented in this paper).

At present, it is possible to turn up and turn down the power consumption in buildings with low control latency using an external control signal. Nevertheless, in future work, estimation of the time duration up to which hold of these changed power levels provides the estimated energy flexibility at that moment. Estimating the heat stored in buildings can inform us about the time deration it can sustain the comfort levels with altered power levels. In different approaches, we can use outdoor temperature trends, indoor condition and occupants’ situation to forecast the energy flexibility better in order to develop this concept further.

However, the current system has some limitations. First, the non-z wave devices (i.e., HTTP) can only be added manually and it is one-time manual task and second the system operation is limited to provide power flexibility. However, in future work there is a plan to develop machine-learning tools to estimate energy flexibility available at any moment in real time. Therefore, the developed BEMS tool opens a future path for energy flexibility estimation in buildings and way to offer it directly on 1-tier double auction, energy flexibility marketplace at zero trading cost by using software based trading bots and subsequently bypassing the need of flexibility aggregators or sub-aggregators. 

Moreover, electric vehicles, photovoltaics and battery storage will have their own positive impact on reducing the carbon content of power systems by increasing the consumption of electricity with high renewable share but there will be need to utilize these local resources very smartly. This presented BEMS work is to better understand the building’s thermal loads and their smart load management is first step towards it. After this success in flexible thermal load management; in future electric vehicles, photovoltaics and battery storage are also to be added in this BEMS software system and then based on electricity price information, local production, thermal flexibility and battery capacity we can make smarter decision to maximize the local consumption of local energy production. For example, morning peak load can be managed by battery storage, evening peak can be reduced by heat pump flexibility, and electric vehicle and battery storage can be charged during access PV production or in mid night during power duck duration. In future, there will be use of this BEMS system to test different control scenarios and also to model building loads to understand the best options for thermal energy flexibility trading using software bots.

## Figures and Tables

**Table 1:** Flexible device status after applying different control actions.

Control Type	Heat Pump	Heater	Floor Heater
Min. Power	off	off	off
Max. Power	on, set point: previous, mode: previous	on	on
Cooling	on, set point: 17 °C, mode: cooling	off	off
Heating	on, set point: 23 °C, mode: heating	on	on

## Data Availability

The study did not publish any public data.

## Abbreviations

The following abbreviations are used in this manuscript:

4G	Fourth generation broadband cellular network technology
AC	Air conditioner
ACs	Air conditioners
API	Application program interface
BEMS	Building energy management system
BB	Beagle board
BLE	Bluetooth low energy
CAN	Controller area network
CO_2_	Carbon dioxide
DC	Direct current
ESP	Espressif systems
EU	European Union
FDP3	FDP3-Modbus
FLEXIMAR	FLEXIMAR—Novel marketplace for energy flexibility
GPIO	General purpose input output
GtCO_2_	Gigatonnes of carbon dioxide
GW	Gateway
HTTP	Hypertext transfer protocol
IEA	International energy agency
ICT	Information and communication technologies
IoT	Internet of things
LAN	Local area network
M2M	Machine-to-machine communication
MQTT	Message queuing telemetry transport
Pdiff	Air pressure difference
PIR	Passive infrared sensor
PLC	Programmable logic controller
PV	Photovoltaics
Rpi	Raspberry pi
SULPU	Suomen lämpöpumppuyhdistys (Finnish Heat Pump Association)
TCP/IP	Transmission control protocol/Internet protocol
THP	Temperature humidity pressure
VTT	Teknologian tutkimuskeskus VTT Oy (VTT Technical Research Centre of Finland Ltd.)

## References

[1] Energy Consumption in Households—Statistics Explained. https://ec.europa.eu/eurostat/statistics-explained/index.php/Energy_consumption_in_households#Energy_consumption_in_households_by_type_of_end-use.

[2] Barberi P., Bossmann T., Fournié L. (2018). Decentralised Heat Pumps: System Benefits under Different Technical Configurations. https://ec.europa.eu/energy/sites/ener/files/documents/metis_s6_-_decentralised_heat_pumps_-_final_report.pdf.

[3] Tracking Buildings 2020—Analysis—IEA. https://www.iea.org/reports/tracking-buildings-2020.

[4] The Future of Cooling—Analysis—IEA. https://www.iea.org/reports/the-future-of-cooling.

[5] Attar M. (2019). Distribution System Congestion Management through Market Mechanism. Master’s Thesis.

[6] Esther B.P., Kumar K.S. (2016). A survey on residential Demand Side Management architecture, approaches, optimization models and methods. Renew. Sustain. Energy Rev..

[7] Mataloto B., Ferreira J.C., Cruz N. (2019). LoBEMS—IoT for Building and Energy Management Systems. Electronics.

[8] Building Energy Management Systems BEMS—Designing Buildings Wiki. https://www.designingbuildings.co.uk/wiki/Building_energy_management_systems_BEMS.

[9] Vašak M., Starčić A., Lešić V., Martinčević A. Upgrade of a typical office building automation system for enabling open energy management services. Proceedings of the International Conference on Smart Systems and Technologies 2017, SST.

[10] Na U., Lee E.-K. (2020). Fog BEMS: An Agent-Based Hierarchical Fog Layer Architecture for Improving Scalability in a Building Energy Management System. Sustainability.

[11] Motlagh N.H., Khajavi S.H., Jaribion A., Holmstrom J. An IoT-based automation system for older homes: A use case for lighting system. Proceedings of the IEEE 11th International Conference on Service-Oriented Computing and Applications, SOCA.

[12] Soetedjo A., Nakhoda Y.I., Saleh C. (2019). An Embedded Platform for Testbed Implementation of Multi-Agent System in Building Energy Management System. Energies.

[13] Market Overview—EHPA. https://www.ehpa.org/market-data/market-overview/.

[14] Suomen lämpöpumppuyhdistys, In Finland Another Peak Year for Heat Pumps. More than 100,000 Pumps Were Sold. https://www.sulpu.fi/documents/184029/0/Pressrelease%2CSULPU%2C1.2020_en.pdf.

[15] Liu Z., Wu Q., Petersen P. Final Report Final Report D2.1. Large Scale Deployment of Electric Vehicles (EVs) and Heat Pumps (HPs) in the Nordic Region. www.cee.dtu.dk.

[16] Heljo J., Harsia P., Holttinen H., Aalto P., Björkqvist T., Järventausta P., Kaivo-Oja J., Kojo M., Korpela T., Rautiainen A. (2016). The January Power Peak-What Happened on 7.1.2016? How to Manage Power Better in the Future?. www.el-tran.fi;@Eltranteam.

[17] Jaaskelainen J., Zakeri B., Syri S. Adequacy of power capacity during winter peaks in Finland. Proceedings of the International Conference on the European Energy Market, EEM.

[18] Fischer D., Surmann A., Lindberg K.B. (2020). Impact of emerging technologies on the electricity load profile of residential areas. Energy Build..

[19] Fischer D., Madani H. (2017). On heat pumps in smart grids: A review. Renew. Sustain. Energy Rev..

[20] Fischer D. (2017). Integrating Heat Pumps into Smart Grids. Ph.D. Thesis.

[21] Fischer D., Wolf T., Triebel M.-A. Flexibility of heat pump pools: The use of SG-Ready from an aggregator’s perspective. Proceedings of the 12th IEA Heat Pump Conference.

[22] Kensby J., Trüschel A., Dalenbäck J.O. (2015). Potential of residential buildings as thermal energy storage in district heating systems—Results from a pilot test. Appl. Energy.

[23] Reynders G., Nuytten T., Saelens D. (2013). Potential of structural thermal mass for demand-side management in dwellings. Build. Environ..

[24] le Dréau J., Heiselberg P. (2016). Energy flexibility of residential buildings using short term heat storage in the thermal mass. Energy.

[25] Cooling—Analysis—IEA. https://www.iea.org/reports/cooling.

[26] Global Cooling Prize. https://globalcoolingprize.org/.

[27] Do Residential AC Buyers Prioritise Energy Efficiency? | CEEW. https://www.ceew.in/publications/do-residential-ac-buyers-prioritise-energy-efficiency.

[28] The Future of Cooling in China—Analysis—IEA. https://www.iea.org/reports/the-future-of-cooling-in-china.

[29] Waite M., Cohen E., Torbey H., Piccirilli M., Tian Y., Modi V. (2017). Global trends in urban electricity demands for cooling and heating. Energy.

[30] Gaur K., Kumar H., Agarwal R.P.K., Baba K.V.S., Soonee S.K. Analysing the electricity demand pattern. Proceedings of the 2016 National Power Systems Conference, NPSC.

[31] Air Conditioning Use Emerges as One of the Key Drivers of Global Electricity-Demand Growth—News—IEA. https://www.iea.org/news/air-conditioning-use-emerges-as-one-of-the-key-drivers-of-global-electricity-demand-growth.

[32] Ala G., di Gangi A., Zizzo G. A methodology for evaluating the flexibility potential of domestic air-conditioning systems. Proceedings of the 20th IEEE Mediterranean Electrotechnical Conference, MELECON.

[33] Demand Response—Analysis—IEA. https://www.iea.org/reports/demand-response.

[34] Perspectives for the Clean Energy Transition—Analysis—IEA. https://www.iea.org/reports/the-critical-role-of-buildings.

[35] Immonen A., Kalaoja J. (2019). Requirements of an Energy Data Ecosystem. IEEE Access..

[36] Tampere University of Technology, Tampere University of Applied Sciences, Lappeeranta University of Technology (2015). Demand Response—Practical Solutions and Impacts for DSOs in Finland.

[37] FLEXIMAR. http://www.fleximarex.com/.

[38] Wall Plug—Smart Outlet with Power Metering | FIBARO. https://www.fibaro.com/en/products/wall-plug/.

[39] RaZberry—Z-Wave.Me. https://z-wave.me/products/razberry/.

[40] Määttä K., Rehu J., Tanner H., Känsälä K. Building intelligence—Home operating system for smart monitoring and control. Proceedings of the IEEE International Conference on Electro Information Technology.

[41] Components & Attributes of Oulu Smart Grid Laboratory (VTT)—ERIGrid. https://erigrid.eu/components-attributes-of-oulu-smart-grid-laboratory-vtt/.

[42] The Internet of Things with ESP32. http://esp32.net/.

[43] Z-Way-Manual. https://github.com/Z-Wave-Me/Z-Way-Manual/blob/master/ZWayManual.pdf.

[44] Z-Wave.Me Team Z-Way Developers Documentation. https://storage.z-wave.me/docs/zwayDev-v2.1.pdf.

[45] Z-Way API·Apiary. https://zwayhomeautomation.docs.apiary.io/#.

